# Performance and Statistical Assessment of Deep Learning Frameworks for Low-Temperature Selective Catalytic Reduction of NO_*x*_

**DOI:** 10.1021/acsomega.6c01308

**Published:** 2026-05-25

**Authors:** Himmet Özarslan, Ihsan Uluocak, Evrim Köylüoǧlu, Kerimcan Çelebi, Ali Keskin

**Affiliations:** † Department of Mechanical Engineering, Faculty of Engineering and Architecture, Kahramanmaras Sutcu Imam University, Kahramanmaras 46100, Turkiye; ‡ Department of Mechanical Engineering, Ceyhan Engineering Faculty, 37506 Cukurova University, Adana 01950, Turkiye; § Department of Automotive Engineering, Engineering Faculty, 37506 Cukurova University, Adana 01950, Turkiye

## Abstract

Nitrogen oxides (NO_*x*_) are among the most harmful emissions produced by internal combustion engines, contributing to air pollution, acid rain, and climate-related impacts. Accurate modeling of the NO_*x*_ reduction process is therefore essential for developing efficient emission control technologies and optimizing catalytic performance. This study explores the potential of advanced deep learning architectures to predict NO_*x*_ conversion efficiency in an ethanol-assisted selective catalytic reduction (SCR) system. Long Short-Term Memory (LSTM), Temporal Convolutional Network (TCN), and a hybrid TCN-LSTM were designed and optimized using Bayesian optimization to obtain the most effective hyperparameter configuration. Experimental data collected from a two-cylinder diesel engine equipped with Ag–Sn–P-based catalysts were used for model training and validation. Model performance was evaluated through Mean Absolute Error (MAE) and Theil’s U_2_ indicators, supported by bootstrap confidence interval analysis to ensure statistical robustness. Among the tested models, the TCN achieved the best predictive accuracy, with a mean absolute error of 0.118 and a Theil’s U_2_ value of 0.051, followed closely by the hybrid TCN-LSTM model with an MAE of 0.132 and U_2_ of 0.053. The results confirm that convolutional and hybrid architectures provide reliable and generalizable frameworks for capturing the nonlinear dynamics of catalytic NO_*x*_ reduction, offering valuable insights for next-generation emission control strategies.

## Graphical abstract

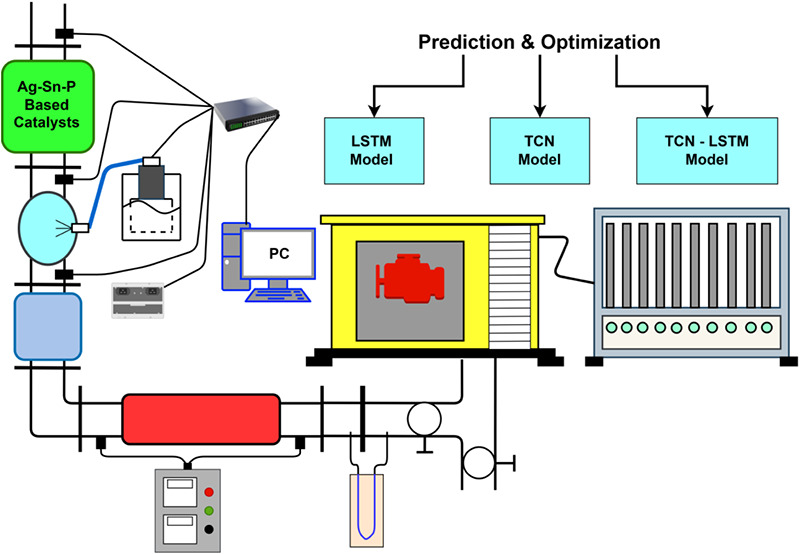

## 1 Introduction

Nitrogen oxides (NO_*x*_), primarily nitric oxide (NO) and nitrogen dioxide (NO_2_), are among the most harmful gaseous pollutants released from diesel engines, contributing to photochemical smog, acid deposition, ozone depletion and severe cardiopulmonary diseases.^1^ Due to increasingly stringent emission regulations, efficient aftertreatment systems have become essential to reduce NO_*x*_ under oxygen-rich exhaust conditions typical of diesel combustion. Selective Catalytic Reduction (SCR) is widely recognized as one of the most effective NO_*x*_ abatement technologies, where gaseous NO_*x*_ is reduced to environmentally benign nitrogen (N_2_) and water (H_2_O) in the presence of a catalyst and a suitable reductant.^2^ Depending on the reductant used, SCR systems are mainly categorized as Ammonia Selective Catalytic Reduction (NH_3_–SCR), Hydrocarbon Selective Catalytic Reduction (HC-SCR), Hydrogen Selective Catalytic Reduction (H_2_–SCR) or Carbon Monoxide Selective Catalytic Reduction (CO-SCR).^2,3^

Among these De-NO_*x*_ technologies, NH_3_–SCR is currently the most commercially implemented method, particularly in automotive and stationary diesel engine applications, owing to its superior NO_*x*_ conversion efficiency over a broad temperature range. However, the practical implementation of NH_3_–SCR systems, where ammonia is typically generated from urea decomposition, suffers from several operational drawbacks.^3^ These include ammonia slip, urea crystallization at temperatures below −11.4 °C, and the requirement for complex urea storage and dosing infrastructure.^4,5^ Furthermore, the catalysts used in these systems exhibit significant sensitivity to phosphorus and sulfur poisoning present in real exhaust gases, leading to a loss of catalytic activity.^4^ The corrosive nature of ammonia and the high operational costs associated with urea supply have further motivated the search for alternative technologies.^5^

As a compelling alternative, HC-SCR has emerged as a promising approach. The primary advantage of HC-SCR over NH_3_–SCR is its ability to use unburned hydrocarbons or the diesel fuel itself as the reducing agent, thereby eliminating the need for an additional ammonia or urea source and simplifying the overall aftertreatment system. Additionally, HC-SCR allows for the simultaneous oxidation of unburned hydrocarbons and carbon monoxide while reducing NO_*x*_ emissions.^3^ Despite these practical benefits, HC-SCR typically exhibits significantly diminished conversion efficiency in relation to the highly optimized NH_3_–SCR process, particularly under lean-burn conditions.^2,3^ Its main limitations include low de-NO_*x*_ activity at temperatures below 250 °C and a narrow operating temperature window, which currently restrict its widespread practical application.^2^

Selective catalytic reduction of NO_*x*_ with hydrocarbons (HC-SCR) serves as a promising alternative to NH_3_–SCR, which is limited by ammonia slip and the technical complexity of urea dosing systems.^6,7^ Among various reductants, ethanol has gained prominence as a high-efficiency, bioderived agent.^8^ Unlike long-chain alkanes, ethanol’s oxygenated structure promotes the formation of active surface intermediates, specifically “enolic” species and acetates, which are crucial for the reduction of NO_*x*_ to N_2_ via isocyanate (−NCO) pathways.^9,10^

Previous research has established that silver-based catalysts exhibit high activity for ethanol-SCR, where the intimate contact between enolic species and active metal sites significantly enhances de-NO_*x*_ efficiency.^10^ Mechanistic investigations have shown that ethanol provides a lower activation energy for NO_*x*_ reduction compared to typical hydrocarbons.^11^ Moreover, ethanol-SCR systems have demonstrated robust performance in real heavy-duty diesel engine applications, maintaining efficiency even under complex exhaust gas compositions.^12^ Recent efforts have focused on steering the reactivity of these systems by adding multifunctional promoters like WO_*x*_ or modifying support architectures to expand the operating temperature window.^13,14^ In this context, the synergy between ethanol and SnO_2_-modified TiO_2_ systems, particularly under varying engine loads, represents a critical area for optimizing NO_*x*_ abatement strategies.^15,16^

Consequently, there is a critical necessity for research focused on developing novel catalysts capable of achieving high NO_*x*_ conversion at lower temperatures to bridge this performance gap and make HC-SCR a viable successor to conventional systems.

Among HC-SCR catalysts investigated, Ag/Al_2_O_3_ has shown the most promising performance owing to its strong hydrocarbon activation capability and high redox stability.^2,17^ For instance, 1 wt % Ag/Al_2_O_3_ catalysts achieve approximately 85% NO_*x*_ conversion around 540 °C,^2^ while dual Ag–Pd configurations further increase conversion efficiencies to ∼90% in the 300–600 °C range.^3^ Additional studies incorporating CuSn-ZSM-5 into Ag/Al_2_O_3_ structures reported >85% NO_*x*_ conversion under similar conditions.^6^ A multifunctional AgW/Al_2_O_3_ catalyst (4 wt % Ag and 6 wt % W) has been reported to achieve approximately 95% NO_*x*_ conversion to N_2_ at 350 °C when using ethanol as a reductant. This catalytic system maintains superior reactivity across a broad temperature range of 200–500 °C compared to the bare Ag/Al_2_O_3_ catalyst. However, Ag/Al_2_O_3_ exhibits limited activity below 250 °C and is prone to sulfur poisoning, motivating the need for catalytic promoters.

Tin (Sn) has been reported to improve both the redox cycle and surface oxygen vacancy concentration, enhancing NO activation and increasing low-temperature conversion efficiency.^15,18^ Specifically, the addition of Fe and Mn dopants to CeO_2_–SnO_2_/Al_2_O_3_ nanocomposites has been shown to create structural defects that enhance oxygen ion mobility, with Mn-doped samples reaching up to 92% NO reduction efficiency.^39^ Sn-containing systems such as Sn-modified Mn–Ce oxides achieved nearly 100% NO_*x*_ conversion in the 110–230 °C range,^17^ while Sn–Ce–Ti-based catalysts maintained ∼94–95% conversion between 200–400 °C.^18,22,23^ Furthermore, Sn modification significantly improves both the NH_3_–SCR activity and hydrothermal stability of CeO_2_-based catalysts; for instance, the Ce_1_Sn_2_Nb_1_O_*x*_ catalyst maintained over 90% NO_*x*_ conversion in the 325–500 °C range even after hydrothermal aging at 1000 °C.^40^ In HC-SCR, Sn-modified Ag-based systems demonstrated marked improvements as well, with Ag–Sn/γ-Al_2_O_3_ achieving ∼88% NO_*x*_ conversion near 400 °C and >80% between 336–448 °C.^14^ Moreover, in CeVO_4_/SnO_2_ systems, the use of SnO_2_ increases Lewis acidity to facilitate NH_3_ adsorption, and formulations containing sulfated SnO_2_ exhibit high SO_2_ resistance with over 90.0% NO_*x*_ conversion between 240–430 °C.^41^ Likewise, SnO_2_/Al_2_O_3_ and SnO_2_/Beta formulations achieved ∼78–83% conversion between 250–450 °C, depending on dispersion effects and support morphology.^15,16^ Regarding preparation methods, studies on impregnation sequences revealed that Ce–Nb/SnO_2_ catalysts prepared by loading Nb before Ce yield superior performance, highlighting that the robust hydrothermal stability of SnO_2_-based materials is critical for diesel vehicle applications.^42^ These results collectively indicate that Sn promotes oxygen vacancy formation and widens the low- and mid-temperature operating window.

Similarly, phosphorus (P), once considered a poisoning species, has recently been shown to significantly improve catalyst durability when introduced in optimized amounts by stabilizing active metal dispersion, increasing Brønsted acidity, and improving SO_2_/H_2_O tolerance.^19–21^ P-modified V_2_O_5_–WO_3_–TiO_2_ catalysts maintained >90% NO_*x*_ conversion within 350–450 °C while preserving long-term structural stability.^19,20^ In addition, P-doped Ce–Ti catalysts sustained ∼88–90% conversion across 200–350 °C, demonstrating its role in enhancing redox stability and structural resistance to deactivation.^5,21^ Furthermore, a series of CePO_4_ modified V_2_O_5_–WO_3_–CeO_2_/TiO_2_ catalysts showed that the incorporation of phosphate significantly increased Brønsted acid site density and chemisorbed oxygen content, leading to a T_90_ as low as 205–207 °C and superior water resistance with only a 4.8% drop in activity under 10–20% H_2_O at 180 °C.^43^ Additionally, the performance of CeO_2_/TiO_2_ catalysts is highly sensitive to the stage of phosphorus introduction, where adding P before loading Ce species boosts low-temperature activity significantly (achieving 98% NO_*x*_ removal at 240 °C) by promoting the dispersion and anchoring of active Ce species on the support.^44^ These findings demonstrate the complementary nature of Ag, Sn and P in extending catalytic activity across the temperature spectrum while enhancing structural resilience.

In recent years, deep learning has emerged as a powerful computational framework for modeling complex nonlinear systems that are difficult to describe through conventional physical or empirical equations. This capability has made deep learning particularly attractive in engineering applications such as combustion optimization,^24^ catalyst performance evaluation,^25,26^ and emission control,^27–29^ where multiple interacting variables influence system responses in a highly nonlinear manner. By leveraging architectures which are used in our study, Long Short-Term Memory (LSTM), Temporal Convolutional Networks (TCN), and their hybrid combinations, researchers have achieved significant improvements in both prediction accuracy and generalization,^30,31^ paving the way for data-driven approaches that complement and extend conventional modeling techniques.

Despite the individual benefits of Ag, Sn, and P modified catalysts reported in the literature, their synergistic combination within a single HC-SCR catalyst structure has not been extensively explored, especially under practical diesel exhaust conditions. The rationale for examining this specific ternary combination lies in the need to simultaneously address the three primary hurdles of HC-SCR: low-temperature ignition, oxygen mobility, and surface acidity stability. While Ag serves as the primary active phase for hydrocarbon activation, it often suffers from a narrow temperature window and poor stability. In this study, Sn is integrated specifically to enhance redox cycles and oxygen vacancy formation, thereby promoting NO activation, while P is incorporated to modulate surface acidity and provide structural resilience against deactivation. Therefore, the objective of this research is to investigate how the synergistic interaction between these three components can overcome the inherent performance trade-offs of single-component catalysts. To this end, ternary Ag–Sn–P-based catalysts are evaluated under realistic diesel engine exhaust conditions, rather than synthetic gas mixtures. The experiments are performed across a critical low-temperature regime (170–270 °C) at various engine loads (1 kW, 3 kW, and 5 kW) and a gas hourly space velocity GHSV of 30,000 h^–1^, providing a comprehensive assessment of the catalyst’s direct applicability for diesel aftertreatment systems.

## 2 Materials and Methods

### 2.1 Catalyst Preparation

Cordierite (2Al_2_O_3_·5SiO_2_·2MgO) was employed as the monolithic support due to its favorable thermo-mechanical properties, including a fine pore structure (average pore diameter ≈ 18 μm), high thermal resistance, and low surface area (∼0.5 m^2^ /g).^32^ Since its intrinsic surface area is insufficient for effective catalytic dispersion, a pretreatment step was applied to improve the surface textural properties.

For this purpose, the cordierite substrates were immersed in a solution containing 500 mL of distilled water and 250 mL of oxalic acid and kept under static soaking conditions for 4 h to promote surface etching. Following impregnation, the substrates were thoroughly rinsed with distilled water until no residual acid remained. The neutralized samples were then dried in a convection oven at 120 °C for 3 h and subsequently calcined in a muffle furnace at 550 °C for an additional 3 h to stabilize the modified surface before coating.

The compositional ratios of the catalytic formulations and the corresponding synthesis pathway are listed in Table 1. The first catalyst (APT) was fabricated as a 50 g mixture containing 2.75 wt % Ag (1.375 g), 1 wt % P (0.5 g), and 96.25 wt % TiO_2_ (48.125 g). To ensure the targeted Ag loading, 2.1653 g of AgNO_3_ was accurately weighed and homogenized with 48.125 g of TiO_2_. The powder mixture was dispersed in 200 mL of deionized water and magnetically agitated for approximately 1 h under mild heating to promote solvent evaporation. As the water content progressively decreased, the suspension transitioned into a dense slurry, which was subsequently transferred to a drying oven and held at 130 °C for 3 h to ensure complete dehydration. The dried material was carefully detached from the vessel, placed in a porcelain crucible, and calcined in a muffle furnace at 550 °C for 3 h to obtain the active catalytic powder.

**Table 1:** Rate of Compositions of the Prepared Catalysts

		Material amounts			
Catalysts	Abbreviation of Catalysts	AgNO_3_	(NH_4_)(H_2_PO_4_)	SnO_2_	TiO_2_
Ag–P/TiO_2_	APT	2.75% = 1.375 g	1% = 0.5 g	-	96.25% = 48.125 g
Ag–P–Sn(1%)/TiO_2_	APSn1T	2.75% = 1.375 g	1% = 0.5 g	1% = 0.5 g	95.25% = 47.625 g
Ag–P–Sn(2%)/TiO_2_	APSn2T	2.75% = 1.375 g	1% = 0.5 g	2% = 1 g	94.25% = 47.125 g

Postcalcination, the powder was finely milled and combined with 1 wt % SiO_2_ (relative to the catalyst mass) to improve adhesion to the cordierite monolith. The resulting mixture was suspended in 400 mL of deionized water and stirred for 1 h at ambient conditions to prepare the coating slurry. Acid-pretreated cordierite substrates were subsequently immersed in the slurry via a dip-coating procedure, which was performed twice, with intermediate drying at 130 °C for 1 h. After the final coating step, the samples were calcined once more at 550 °C for 3 h, yielding the APT catalyst-coated monolith.

Two other catalyst formulations were produced following the same synthesis protocol. In the second catalyst (APSn1T), 1 wt % Sn (0.5 g SnO_2_) was introduced, yielding a final composition of 2.75 wt % Ag, 1 wt % P, 1 wt % Sn, and 95.25 wt % TiO_2_. For the third catalyst (APSn2T), the Sn loading was increased to 2 wt % (1 g SnO_2_). Except for the modified precursor ratios, the successive stages of dissolution, mixing, drying, calcination, milling, and dip-coating were identically replicated across all formulations to maintain reproducibility and comparability.

### 2.2 SCR Performance Test System

The experimental setup is based on an AKSA A2CRX08 diesel engine, configured with two cylinders and operating at a fixed speed of 3000 rpm. The unit is equipped with an 80 mm bore and a 79 mm stroke, providing a cylinder volume of 830 cm^3^ and a compression ratio of 23:1. Engine cooling is achieved through a water-based thermal management system to ensure stable operation under sustained loading conditions.

For emission control, the engine is integrated with a post-treatment assembly comprising a loading system, a microprocessor unit, an exhaust gas heater, a selective catalytic reduction (SCR) catalyst, and a diesel oxidation catalyst (DOC). Ethanol was employed as the reductant and was supplied to a dedicated mixing chamber located between the SCR and DOC through a six-point electro-hydraulic injector driven by an electric pump. Exhaust gas flow rates were quantified using an orifice plate, while pressure values were monitored via a U-tube manometer.

Engine loading was achieved using a resistive heating system consisting of heater elements, each rated at 1 kW, enabling incremental adjustment of the applied load in 1 kW steps. By selectively engaging the heaters, the desired engine load levels were imposed in a controlled manner. This approach enabled systematic variation of operating conditions while maintaining stable thermal behavior, thereby facilitating a consistent assessment of catalyst performance across different load regimes.

Temperature control within the exhaust line was managed through K-type thermocouples, capable of operating between −200 and 1370 °C, which provided feedback to an electric heater regulating the gas stream in the 170–270 °C range required for testing. The exhaust routing incorporated a set of control valves and an orifice plate dimensioned to achieve a space velocity of approximately 30,000 h^–1^, followed by a diesel oxidation catalyst prior to the reductant injection zone.

For emissions monitoring, the setup employed a pair of Continental UniNOx sensors, placed before and after the SCR unit to measure NO_*x*_ concentrations at both inlet and outlet streams. The sensor outputs were transferred to a data acquisition platform via a CANBUS Shield communicating with an Arduino Due microcontroller, enabling continuous real-time logging of exhaust composition. For the reductant delivery line an SRD-05VDC-SL-C controlled pump maintained precise 3–3.5 bar pressure to a 6-hole injector. This regulated pressure ensured effective atomization of the ethanol through the six-orifice injector, promoting uniform mixing with the exhaust gases.

All experimental runs were carried out under standardized conditions to ensure reproducibility. Prior to data acquisition, the engine was preheated for approximately 30 min to reach a thermally stable state. Each catalyst formulation was examined under a fixed space velocity of 30,000 h^–1^ within the operational temperature interval of 170–270 °C. The influence of engine loading on catalytic performance was investigated by subjecting the system to three discrete load levels; 1 kW, 3 kW, and 5 kW. Load variation was accomplished in 1 kW increments through selective activation of resistive heating elements, allowing for precise modulation of the applied mechanical demand. This procedure enabled a controlled transition from light (1 kW) to moderate (5 kW) load conditions while preserving steady exhaust flow and temperature characteristics, thereby providing a consistent basis for evaluating conversion efficiency across the tested catalysts.

### 2.3 Methodology

In this study three distinct modeling is approached to predict the NO_*x*_ reduction efficiency of various catalyst configurations. The methods include Long Short-Term Memory (LSTM) networks, Temporal Convolutional Networks (TCN), and a hybrid TCN-LSTM model.

Traditional kinetic models for Selective Catalytic Reduction (SCR) processes often rely on complex differential equations that require extensive experimental calibration and significant computational resources. These mechanistic models struggle to account for the highly nonlinear synergies between diverse fuel blends (like ethanol additives), varying catalyst temperatures, and fluctuating engine loads in real-time. Therefore, data-driven predictive models have become a necessity in modern engine management systems. By leveraging deep learning, it is possible to bypass the need for explicit chemical kinetic parameters, providing a more agile and accurate framework for predicting NO_*x*_ conversion efficiency. Such models are vital for developing adaptive control strategies that can respond instantly to transient emission profiles, which is a key challenge for meeting stringent environmental regulations Figure 1.

**Figure 1:**
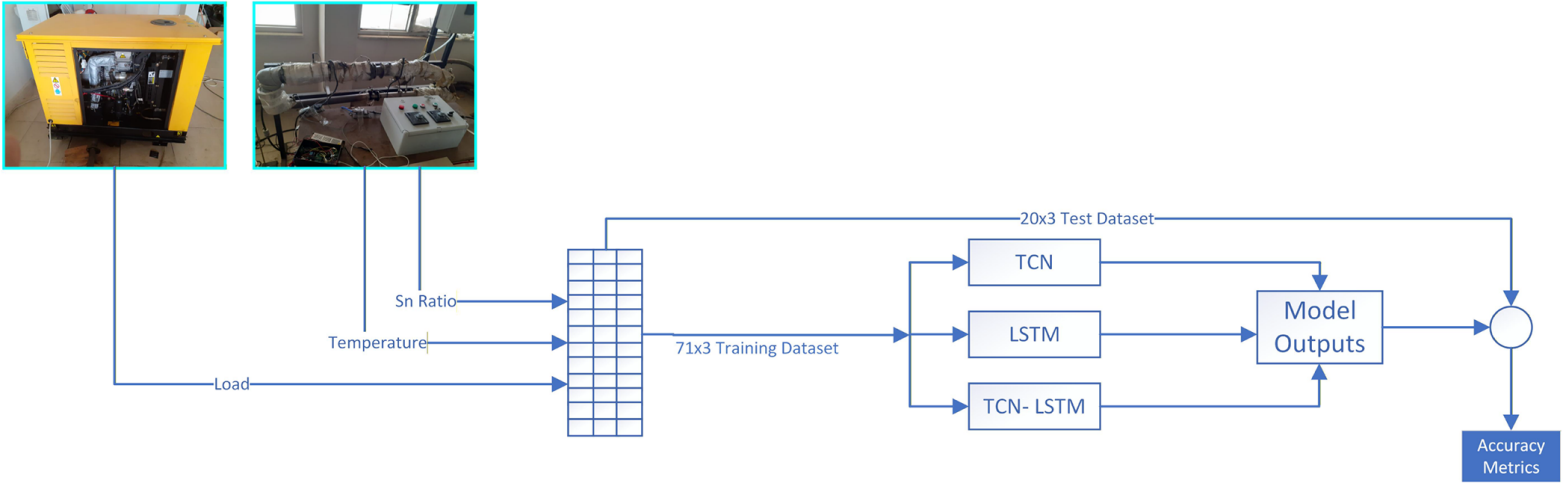
Experiment rig scheme.

The data set utilized in this study comprises 99 experimental data points obtained from various operating conditions. To maximize the utility of the available data and prevent overfitting, a 5-fold cross-validation procedure was adopted. The data was partitioned into five distinct folds, where 80% of the data was used for training and the remaining 20% served as the test set in each rotation. This cycle was repeated five times, ensuring that every data point was used for both training and testing.

#### 2.3.1 LSTM Model

The LSTM network was utilized to capture the temporal dependencies and nonlinear patterns in the data.^33^ Even though our input features are not strictly time-series in the traditional sense, LSTM was chosen for its ability to handle sequences and to learn complex relationships from the input variables (such as Sn ratio, engine load, and exhaust temperature).

#### 2.3.2 TCN Model

Temporal Convolutional Network is also implemented to provide a complementary perspective. TCNs are known for their ability to handle sequential data using causal convolutions and can effectively model long-range dependencies with fewer parameters.^34^ This approach helps in capturing different patterns in the data that LSTM might overlook.

#### 2.3.3 Hybrid TCN-LSTM Model

Finally, we combined the strengths of both TCN and LSTM by developing a hybrid model. In this configuration, the TCN layers were used as a feature extractor to capture local patterns, while the LSTM layers processed the extracted features to learn longer-term dependencies.^35^ This hybrid approach aims to improve the overall predictive accuracy and robustness of the NO_*x*_ emission reduction estimates.

To maximize the predictive performance of the developed models, a Bayesian optimization strategy was implemented. This approach was used to search the hyperparameter space (including learning rate, dropout rate, batch size, and the number of hidden units) over 60 iterations. By minimizing the objective function through this probabilistic model, we ensured that the selected configurations for LSTM, TCN, and the hybrid TCN-LSTM were statistically optimal for the given SCR data set.

The optimal configuration for each model was determined through Bayesian Optimization over the specified ranges (Table 2). Unlike manual tuning, this method allowed for an efficient exploration of the parameter space, ensuring that the hybrid TCN-LSTM model achieved its peak generalization capability (R^2^ = 0.9856) by balancing architectural complexity with the available 99 experimental samples.

**Table 2:** Hyperparameter Optimization Ranges and Their Roles

Hyperparameter	Range	Role in Model Performance
Learning Rate	10^–4^ to 10^–3^	Controls the step size during gradient descent; crucial for convergence stability.
LSTM Units	10 to 100	Determines the memory capacity to capture sequential dependencies in experimental data.
Conv Filters (TCN)	8 to 64	Defines the number of features extracted from input combinations (Temp, Load, Fuel).
Dropout Rate	0.1 to 0.4	Prevents overfitting by randomly deactivating neurons during training.
Mini-Batch Size	4 to 32	Balances the stochastic nature of the gradient estimate and training speed.

#### 2.3.4 Performance Evaluation Metrics

In addition to the modeling approaches, we evaluated the model performance using three key accuracy metrics: Mean Absolute Error (MAE), Theil’s U2 statistic, and Root Mean Squared Error (RMSE). Below are the definitions and formulas for each metric:

Mean Absolute Error (MAE): The MAE measures the average magnitude of the errors between predicted and actual values, without considering their direction. It is calculated as:

Theil’s U2 Statistic: Theil’s U2 is a relative accuracy measure that compares the predictive performance of a model to that of a naive benchmark model.

A U2 value less than 1 indicates that the model is better than the benchmark.

Root Mean Squared Error (RMSE): RMSE is a quadratic scoring rule that measures the average magnitude of the error and is more sensitive to large deviations.

## 3 Results and Discussion

A comprehensive series of experiments was conducted to assess the role of ethanol as a reducing agent and to examine the impact of Ag–P–Sn-based catalytic systems on NO_*x*_ mitigation under diesel exhaust conditions. The catalytic evaluation was performed within a controlled reaction framework to systematically quantify both activity and durability across relevant operating regimes. The resulting measurements were subsequently examined through a detailed interpretation process aimed at clarifying the underlying physicochemical interactions between ethanol and the catalytically active surface sites. Based on these findings, the study formulates research-driven recommendations that emphasize catalyst architecture refinement and the investigation of alternative reductants to improve NO_*x*_ abatement at low temperatures. It should be noted that the measurements were obtained from a single SCR engine setup, which enhances repeatability yet constrains cross-platform verification. Given that the robustness of AI-assisted predictive models is closely linked to the heterogeneity of their training data, future extensions of this work will require data sets acquired from multiple engine configurations and diverse operational environments to achieve broader generalizability.

### 3.1 NO_*x*_ Emission Conversion Rates

Ethanol was employed as the reductant and injected upstream of the APT, APSn1T, and APSn2T catalysts, after which the resulting NO_*x*_ conversion characteristics were examined across a matrix of operating temperatures and engine loads. The experiments were carried out within the temperature window of 170–270 °C (10 °C increments) at a space velocity of 30,000 h^–1^, and under engine loads of 1, 3, and 5 kW. The findings in Figure 2 demonstrate that the catalytic performance exhibited a clear upward trajectory with increasing temperature, indicating enhanced surface reactivity and faster reduction kinetics at elevated thermal conditions. Likewise, higher engine loads consistently promoted NO_*x*_ abatement across all catalyst formulations, implying that increased exhaust mass flow improves reductant–catalyst interaction. The most favorable conversion efficiencies were consistently observed at 270 °C under all tested load conditions, confirming this temperature as the point of maximum conversion within the investigated range. While HC-SCR activity typically trends upward with temperature, this range (170–270 °C) was specifically chosen to match the actual exhaust thermal profile of the test engine under the prescribed load conditions.

**Figure 2:**
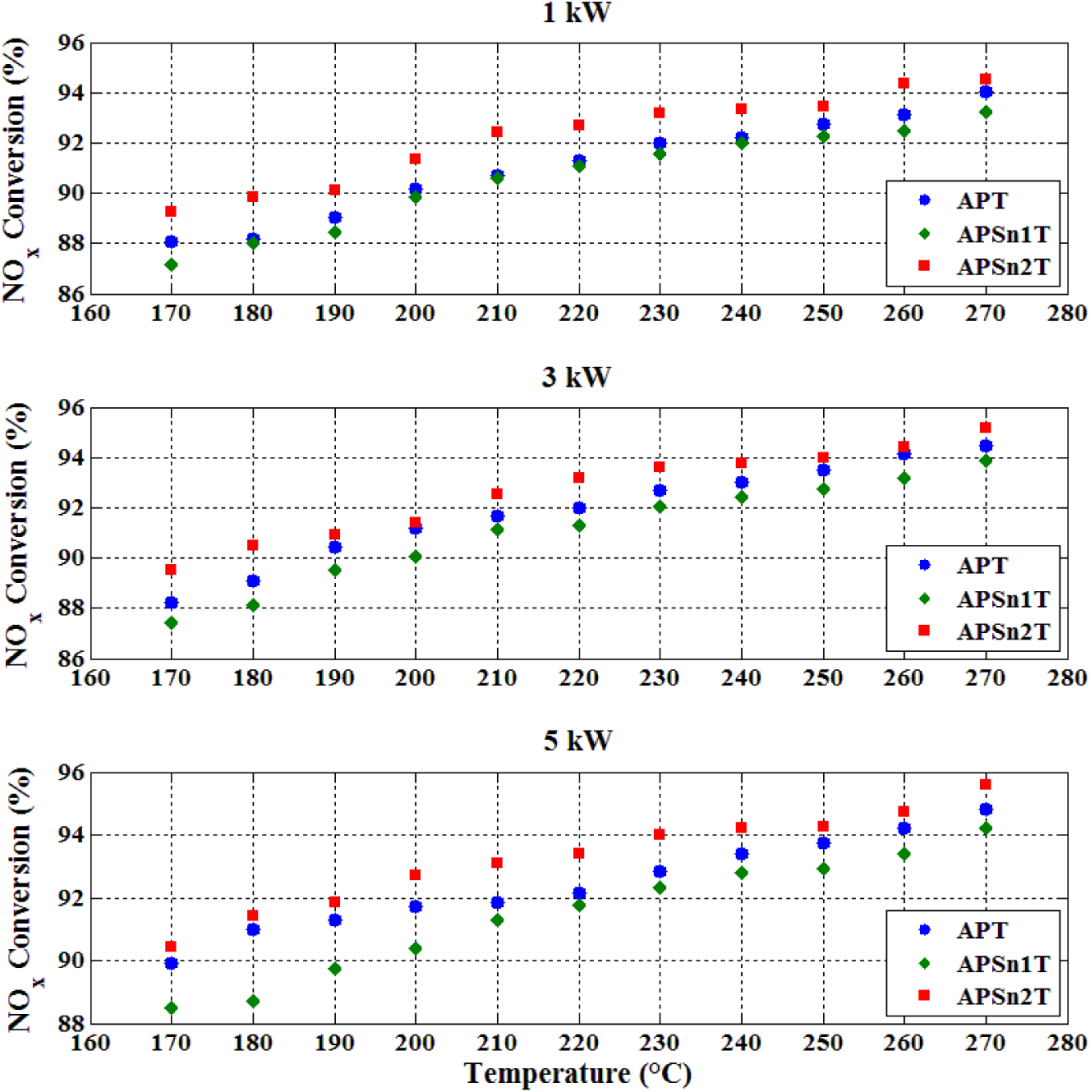
NO*_x_* conversion rates for catalysts at 1 kW, 3 kW and 5 kW, respectively.

Figure 2 illustrates the variation in NO_*x*_ conversion efficiency under different engine loads. As expected, the conversion efficiency increased with engine load, reaching a maximum of 95.58% at 5 kW compared to 87.16% at 1 kW. This improvement is attributed to the higher exhaust temperatures and mass flow rates associated with elevated loads, which enhance thermal activation and surface reaction kinetics.^6,38^ Additionally, the higher load conditions provide a richer supply of hydrocarbon-derived radicals, optimizing the reduction of NO_2_ into N_2_.^36,37^ Across all loads, the peak catalytic activity was consistently observed at 270 °C, confirming the decisive role of temperature in SCR effectiveness.

Under the most favorable operating conditions, namely 270 °C at a 5 kW engine load, the catalytic systems exhibited their peak NO_*x*_ reduction efficiencies, yielding 94.8% for APT, 94.22% for APSn1T, and a slightly higher 95.58% for APSn2T. Conversely, the lowest conversion values were recorded at the lower boundary of the temperature domain (170 °C) under a 1 kW load, where APT achieved 88.05%, APSn1T 87.16%, while APSn2T again performed comparatively better at 89.23%. These findings underscore the pronounced sensitivity of catalyst activity to both thermal conditions and engine load, confirming that elevated temperatures and increased exhaust flow promote more efficient NO_*x*_ reduction across all studied formulations.

The evaluation of Figure 2 reveals that the catalytic performance is significantly influenced by the catalyst composition, specifically the Sn/Ti ratio. It is evident that the APSn2T catalyst, which possesses a higher SnO_2_ content, consistently outperforms both the Sn-free APT and the lower Sn-containing APSn1T across all tested conditions. This enhancement can be attributed to the promotional effect of SnO_2_ on the surface properties of the TiO_2_ support.

According to,^15,16^ the dispersion of SnO_2_ on a support follows a “monolayer dispersion threshold” effect, where reaching an optimal loading significantly increases the concentration of surface acidic sites and oxygen vacancies. In our study, the superior performance of APSn2T suggests that the SnO_2_ loading in this formulation provides a more favorable density of Lewis acid sites, which are crucial for the adsorption and activation of the ethanol reductant and NO_*x*_ species. Furthermore, the integration of Sn into the catalyst structure facilitates the formation of reactive surface intermediates. As demonstrated by,^14^ the synergistic interaction between Sn and the catalyst surface can rationally tailor the redox ability, promoting the conversion of NO to NO_2_ and the subsequent formation of acetate and nitrate species, which are vital for the HC-SCR process. Additionally, Sn modification is known to enhance low-temperature activity by increasing the abundance of active surface oxygen and improving the catalyst’s resistance to bypass reactions. The marginal difference observed between APT and APSn1T suggests that a minimum threshold of Sn is required to fundamentally alter the surface kinetics and achieve the significant efficiency gains seen in the APSn2T formulation.

The results obtained from the optimized deep learning models are presented and discussed in this section. The analysis focuses on evaluating the predictive performance, convergence behavior, and generalization capability of the LSTM, TCN, and hybrid TCN-LSTM architectures in estimating NO_*x*_ conversion efficiency. Both the Bayesian optimization outcomes and the subsequent validation tests are examined to highlight the strengths and limitations of each modeling approach.

The optimized hyperparameters obtained through Bayesian optimization for each model are presented in Table 3. The hybrid TCN-LSTM network achieved stable convergence with an initial learning rate of 0.008, 53 hidden units, 48 filters, a batch size of 28, and a dropout rate of 0.115, providing a good balance between training speed and generalization ability.

**Table 3:** Model Hyperparameters

Model	Initial Learning Rate	Hidden Unit Number	Filter Number	Min Batch Size	Dropout Rate
TCN	0.003	-	60	32	0.105
TCN-LSTM	0.008	53	48	28	0.115
LSTM	0.009	34	-	4	0.318

The pure TCN model reached its optimal configuration with 60 convolutional filters, a learning rate of 0.003, a batch size of 32, and a dropout rate of 0.105, which ensured effective feature extraction and smooth convergence during training.

The LSTM model performed best with an initial learning rate of 0.009, 34 hidden units, a batch size of 4, and a dropout rate of 0.318, reducing overfitting and improving model robustness. Overall, the Bayesian optimization procedure successfully identified efficient and stable parameter combinations that enhanced predictive performance in estimating NO_*x*_ conversion efficiency.

Figure 3 illustrates the correlation between the experimental and predicted NO_*x*_ conversion values obtained by the LSTM, TCN, and hybrid TCN-LSTM models. All data points lie close to the 1:1 reference line, indicating high consistency between model predictions and measured values. The hybrid TCN-LSTM architecture achieves the closest alignment, confirming its superior capacity to generalize nonlinear catalytic behavior under varying operating conditions.

**Figure 3:**
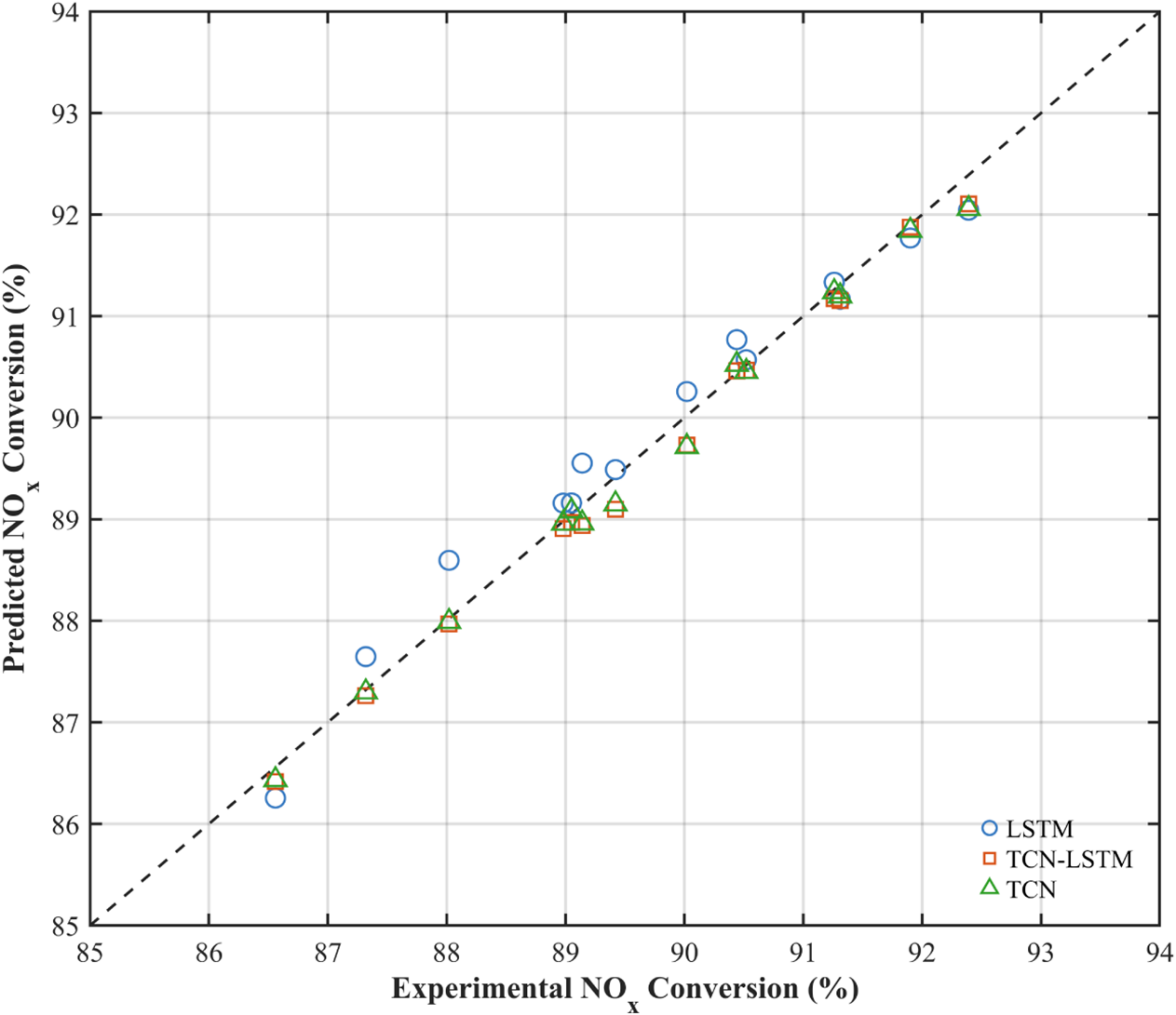
Regression graph for offered models.

Figure 4 compares the experimental NO_*x*_ conversion trend with the predicted responses of each model across all test samples. The LSTM predictions follow the general trend but exhibit slight smoothing around local maxima, while the TCN model reproduces short-term variations more sharply. The hybrid TCN-LSTM model demonstrates nearly perfect phase and amplitude agreement with the experimental data, providing stable, noise-resilient tracking across the entire data set. These visual results collectively verify that integrating convolutional and recurrent structures yields the most accurate and robust representation of NO_*x*_ reduction dynamics.

**Figure 4:**
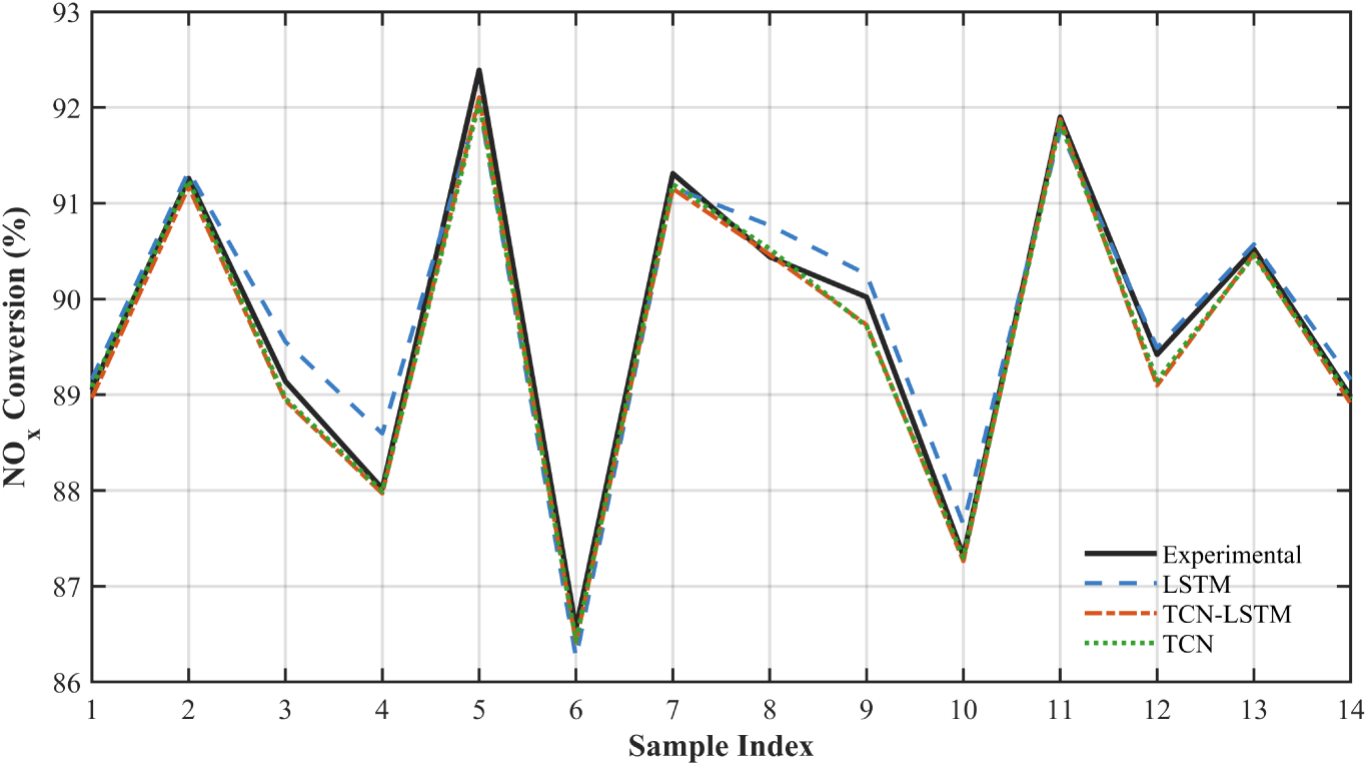
Visual comparison of the prediction models.

Figure 5 presents a comparative evaluation of Theil U_2_, RMSE, and MAE for the LSTM, TCN, and hybrid TCN-LSTM models. Among the tested architectures, the TCN model achieved the lowest error levels, yielding a Theil U_2_ value of approximately 0.051 and a mean absolute error of 0.118, demonstrating its superior predictive accuracy and stability. The hybrid TCN-LSTM model closely followed with a Theil U_2_ of 0.053 and an MAE of 0.132, while the LSTM network recorded the highest error values, with a Theil U_2_ of 0.090 and an MAE of 0.235. These quantitative results indicate that the pure TCN architecture is particularly effective in capturing localized temporal patterns within the catalytic data, whereas the hybrid configuration offers a balanced trade-off between accuracy and generalization. Overall, the findings highlight that convolutional and hybrid deep learning approaches provide robust and highly reliable frameworks for modeling the nonlinear behavior of NO_*x*_ conversion processes.

**Figure 5:**
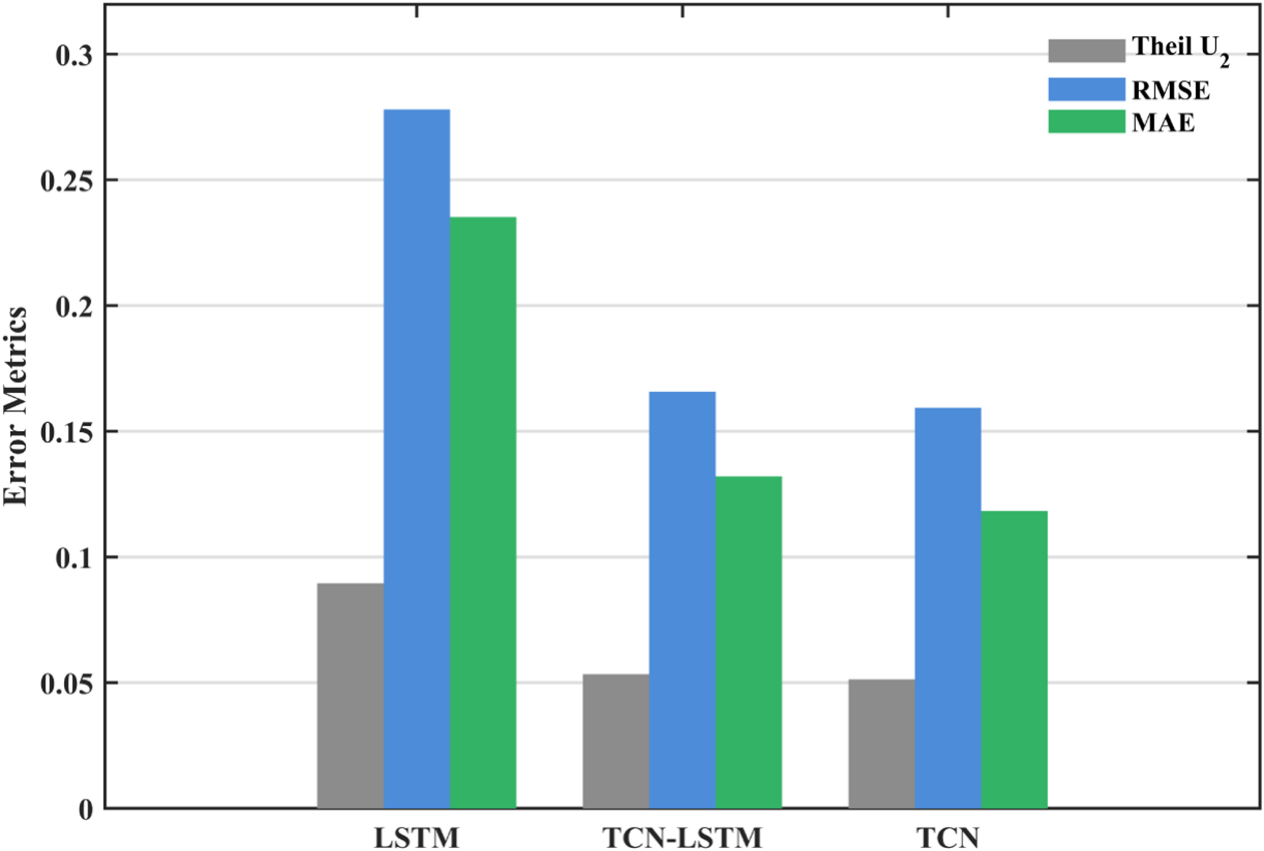
Error metrics results for LSTM, TCN and TCN-LSTM.

A bootstrap resampling analysis was employed to quantify the statistical reliability of the model error estimates and to minimize potential bias arising from limited experimental samples. Using 10,000 iterations, 95% confidence intervals were computed for both the Mean Absolute Error (MAE) and Theil’s U_2_ metrics. The LSTM model showed a mean MAE of 0.235 with a confidence range between 0.16 and 0.32, while the hybrid TCN-LSTM produced a mean MAE of 0.132 within the interval of 0.08 to 0.19. The TCN model achieved the lowest mean MAE of 0.118 with a confidence range from 0.07 to 0.18. The corresponding Theil’s U_2_ values were 0.090 (0.06–0.12) for the LSTM, 0.053 (0.03–0.08) for the TCN-LSTM, and 0.051 (0.03–0.07) for the TCN. The relatively narrow confidence bounds observed for the TCN and TCN-LSTM models indicate greater consistency and lower variance in predictive performance. Overall, the bootstrap analysis confirmed that the improved accuracy of these two models is statistically significant rather than a random outcome, reinforcing their robustness and generalization capability in modeling nonlinear catalytic NO_*x*_ reduction behavior.

## 4 Conclusion

This study demonstrated the effectiveness of deep learning architectures in predicting NO_*x*_ conversion efficiency within an ethanol-assisted SCR system. Bayesian optimization was employed to identify optimal hyperparameters for each network, ensuring stable convergence and improved reliability. Among the tested models, the Temporal Convolutional Network (TCN) achieved the best predictive accuracy, with a mean absolute error of 0.118 and a Theil’s U_2_ value of 0.051. The hybrid TCN-LSTM model produced comparable results, with slightly higher but still low error levels (MAE of 0.132 and U_2_ of 0.053), while the LSTM model showed the largest deviations.

The superior performance of the TCN model highlights the efficiency of convolutional structures in capturing localized temporal dependencies in catalytic processes, whereas the hybrid configuration demonstrated enhanced stability and generalization across varying conditions. These results confirm that convolutional and hybrid deep learning frameworks can serve as powerful tools for modeling nonlinear catalytic NO_*x*_ reduction behavior, providing a data-driven foundation for future intelligent emission control and catalyst optimization strategies.
